# Profiling of lung microbiota discloses differences in adenocarcinoma and squamous cell carcinoma

**DOI:** 10.1038/s41598-019-49195-w

**Published:** 2019-09-06

**Authors:** Sílvia Gomes, Bruno Cavadas, Joana Catarina Ferreira, Patrícia Isabel Marques, Catarina Monteiro, Maria Sucena, Catarina Sousa, Luís Vaz Rodrigues, Gilberto Teixeira, Paula Pinto, Tiago Tavares de Abreu, Cristina Bárbara, Júlio Semedo, Leonor Mota, Ana Sofia Carvalho, Rune Matthiesen, Luísa Pereira, Susana Seixas

**Affiliations:** 1 Instituto de Investigação e Inovação em Saúde, Universidade do Porto (I3S), Porto, Portugal; 2 Institute of Molecular Pathology and Immunology of the University of Porto (IPATIMUP), Porto, Portugal; 3 Pneumology Department, Centro Hospitalar de São João (CHSJ), Porto, Portugal; 4 Department of Pneumology, Unidade Local de Saúde da Guarda (USLG), Guarda, Portugal; 5 Department of Pneumology; Centro Hospitalar do Baixo Vouga (CHBV), Aveiro, Portugal; 6 Unidade de Técnicas Invasivas Pneumológicas, Pneumologia II, Hospital Pulido Valente, Centro Hospitalar Lisboa Norte (CHLN), Lisbon, Portugal; 7 Instituto de Saúde Ambiental, Faculdade de Medicina da Universidade de Lisboa, Lisbon, Portugal; 8 Computational and Experimental Biology Group, CEDOC, Faculdade de Ciências Médicas, Universidade Nova de Lisboa, Lisboa, Portugal; 9 Faculdade de Medicina da Universidade do Porto, Porto, Portugal

**Keywords:** Non-small-cell lung cancer, Microbiome, Chronic obstructive pulmonary disease, Prognostic markers

## Abstract

The lung is a complex ecosystem of host cells and microbes often disrupted in pathological conditions. Although bacteria have been hypothesized as agents of carcinogenesis, little is known about microbiota profile of the most prevalent cancer subtypes: adenocarcinoma (ADC) and squamous cell carcinoma (SCC). To characterize lung cancer (LC) microbiota a first a screening was performed through a pooled sequencing approach of 16S ribosomal RNA gene (V3-V6) using a total of 103 bronchoalveaolar lavage fluid samples. Then, identified taxa were used to inspect 1009 cases from *The Cancer Genome Atlas* and to annotate tumor unmapped RNAseq reads. Microbial diversity was analyzed per cancer subtype, history of cigarette smoking and airflow obstruction, among other clinical data. We show that LC microbiota is enriched in Proteobacteria and more diverse in SCC than ADC, particularly in males and heavier smokers. High frequencies of Proteobacteria were found to discriminate a major cluster, further subdivided into well-defined communities’ associated with either ADC or SCC. Here, a SCC subcluster differing from other cases by a worse survival was correlated with several Enterobacteriaceae. Overall, this study provides first evidence for a correlation between lung microbiota and cancer subtype and for its influence on patient life expectancy.

## Introduction

Lung Cancer (LC) is the most common and lethal cancer worldwide with a reported incidence of 11.6%, a mortality rate of 18.4% and according to recent estimates - 2.1 million new cases^1^. From a histological point of view, non-small cell lung cancer (NSCLC) is the most prevalent type, which can be further subdivided into two major subtypes: adenocarcinoma (ADC) and squamous cell carcinoma (SCC). To date, diverse environmental factors besides cigarette smoking, like biomass burning, indoor and outdoor pollutants, are suggested to play a role in LC pathogenesis, as well as in chronic obstructive pulmonary disease (COPD)^2^. This later illness, characterized as the persistence of airflow limitation in result of bronchitis and/or emphysema, is also recognized as a critical comorbidity in LC, always associated to a worse prognosis^3^. Moreover, a mechanistic link between COPD and LC has been proposed partially due to the findings of chronic inflammation and its repeated cycles of injury and repair, which in COPD are known to promote tumorigenesis and malignant transformation^4^.

Notably and similarly to the model established for *Helicobacter pylori* in gastric cancer, *Mycobacterium tuberculosis* has been hypothesized as a LC agent, once it induces inflammatory markers and causes significant alterations in lung tissues^5,6^. However, this association to tuberculosis is hard to disentangle because of its co-occurrence with other risk factors such as smoking, emphysema and bronchitis^5^.

In this area of knowledge, the impact of lung microbiota, or the bacteria communities inhabiting the lung, has been more extensively studied in COPD than in LC. This is likely to result from COPD patients often suffering from acute exacerbations, which are considered to be of infectious nature and caused by bacteria and/or virus^7^.

Until now, studies aiming to characterize COPD and LC microbiota used different biological specimens, including sputum, bronchoalveaolar lavage fluid (BALF) and lung tissue. And, whereas in COPD, samples were collected during distinct phases of the disease (stable or exacerbation); in LC, those were gathered in some instances from both tumor and non-tumor regions. Moreover, although previous works were mainly based in the screening of 16S ribosomal RNA (16S rRNA) gene, their experimental approaches concerning sample size, hypervariable regions covered and sequencing technologies employed are quite diverse and consequently, their findings are not always consensual^8–20^.

Nonetheless, most studies seem to agree in the common core microbiota of both healthy and diseased subjects dominated by Firmicutes, Bacteriodetes, Proteobacteria and Actinobacteria phyla, and by S*treptococcus*, *Haemophilus*, *Veilonella*, *Pseudomonas* and *Prevotella* genera^8–23^. Interestingly, in COPD, microbiota tends to be relatively stable over time and affected during exacerbations, when it shifts toward Proteobacteria manly due to a *Moraxella* increase and a S*treptococcus* reduction^8,22^. Conversely, in LC, a Firmicutes switch was suggested in result of augmented *Streptococcus*, *Granulicatella*, *Veillonella* and *Megasphaera* prevalence^14,16,21,24^. Critical changes in microbiota composition were suggested to occur along with airway disease progression^12,17,18,22^. However, the use of certain bacterial taxa as potential biomarkers for improved patient stratification or even as treatable traits, is far from being a reality.

Here, we explore an association of lung microbiota with cancer, while addressing also the impact of COPD co-morbidity. Briefly, we performed a microbiota profiling in a small set of Portuguese samples, used later to guide the characterization of bacterial communities in an extended cohort of ADC and SCC cases from *The Cancer Genome Atlas* (TCGA) Research Network^25^. This allowed us to detect significant differences in microbiota diversity of cancer subtypes, as well as to identify among SCC a well-defined community (Enterobacteriaceae) connected to a worse patient survival mainly due to non-cancer complications. Furthermore, we identified *Achromobacter* as a gram-negative bacterium linked with both SCC and COPD.

## Materials and Methods

### Sample collection

BALF was collected from subjects undergoing bronchoscopy for evaluation of lung disease at three hospitals in Portugal: *Centro Hospitalar São João* (CHSJ), in Porto; *Centro Hospitalar Baixo Vouga* (CHBV), in Aveiro; and *Hospital Pulido Valente* - *Centro Hospitalar Lisboa Norte* (CHLN), in Lisbon. Informed consent was obtained for all participants and sample collection for Human Research was approved by hospital ethical committees: *Comissão de Ética para a Saúde* (CES) – CHSJ, *Comissão de Ética* – CHBV and *Comissão de Ética para a Saúde* (CES) – CHLN. The study was conducted in accordance with ethical guidelines and regulations for Human research and with Helsinki Declaration. Sample collection was targeted toward affected lung segments and done by bronchoscope wedging into subsegmental lung regions. In this study, we used only bronchoscope working channel washes, which were done twice with a minimum volume of 15 mL (0.9% saline solution). Samples were then stratified in LC (N = 49) or controls (N = 54) based in positive or negative cytology results (Supplementary Table 1). However, in a follow-up analysis carried out up to 2 years after BALF collection, two cases were found to be false positives and another four initially classified as negative, over time progressed to LC^26^. The pooled sample strategy prevented the reallocation of these cases to controls and vice-versa.

### Lung microbiota 16S rRNA screening

DNA extraction from BALF (200 µL) was performed using DNA Mini kit (Qiagen) according to manufacture instructions for capturing bacterial DNA in body fluids. Two 16S rRNA fragments spanning hypervariable regions V3-V4 and V4-V6 were amplified using universal primers (Supplementary Table 2). Pooled samples containing PCR products (~200 ng/sample) were generated for LC (N = 49) and controls (N = 54) and processed as previously described^27^. Briefly, two libraries were constructed according to Ion Xpress™ Plus Fragment Library Kit protocol (Life Technologies) and ran in an Ion PGM™ System - 316™ chip (Life Technologies). The tools USEARCH, UCHIME, QIIME and Greengenes were used in the analysis of operational taxonomic units (≥97% nucleotide sequence identity cut-off) as previously described^27^.

### TCGA dataset

Raw RNAseq reads from tumors and clinical data files corresponding to 515 ADC and 501 SCC cases from TCGA, were downloaded from Genomic Data Commons (GDC) Data Portal (https://gdc.cancer.gov/). To perform a quantitative analysis of lung microbiota we used reads not aligning with human reference sequence (unmapped reads) as input for QmihR^28^. This pipeline combines Trimmomatic, Bowtie2, and RSEM for a probabilistic inference of bacterial taxa abundances^28^. To instruct bacterial sequence surveys, we first defined a microbiota reference panel based in previous evidence of lung colonization in healthy and diseased patients. Exactly, we considered a total of 567 bacterial taxa according to the data available in: 1) the Human Microbiome Project (HMP) – airways; 2) specialized literature; and 3) our own 16S rRNA study; for which whole genome sequences could be collected from https://www.ncbi.nlm.nih.gov/genome/microbes/. Upon quality control, relative abundances of 112 genera were obtained for 509 ADC and 500 SCC cases. These samples were then stratified according to several variables including ancestry (European or African), gender, age at diagnosis (≤65 or >65), anatomic positioning (Upper or Lower lung), localization in lung parenchyma (Peripheral and Central Lung) and pathological tumor stage (Stages I, II and III + IV). In addition, post-bronchodilator forced expiratory volume in 1 second (FEV1) and forced vital capacity (FVC) ratio were used to determine the presence (FEV1/FVC < 0.70) or absence of airflow obstruction^3^. Smoking history in pack per years (PPY) was considered using a first 20 PPY subdivision (data not shown). Given that most cases largely surpassed this value, a naïve 45 PPY split was used instead based on its proximity to average values (all cases 48; ADC 42 and SCC 53 PPY). Several patient follow-up variables were also considered in this study to evaluate clinical significance of collected data. These included vital (dead or alive) and cancer (tumor free or with tumor) status, days to death and primary therapy outcome.

### Statistical analysis

Statistical analysis of microbiota diversity was performed in R studio (https://www.rstudio.com/; version 1.1.383) using phyloseq^29^. Alpha diversity was evaluated through inverse Simpson and Shannon indexes. Beta diversity, which integrates phylogenetic relationships of bacteria was calculated by weighted UniFrac. Distances matrixes were used in Principal coordinates analysis (PCoA) and in hierarchical clustering (complete linkage) of TCGA samples. The linear discriminant analysis (LDA) effect size (LEfSe) algorithm^30^ was used to detect taxa with differential abundances between TCGA cases. Survival analyses and log rank tests for pairwise comparisons of different case sets were carried out through the *Cohort Comparison* tool available at GDC Data Portal.

## Results

### Characterization of lung bacterial communities

**Figure 1:**
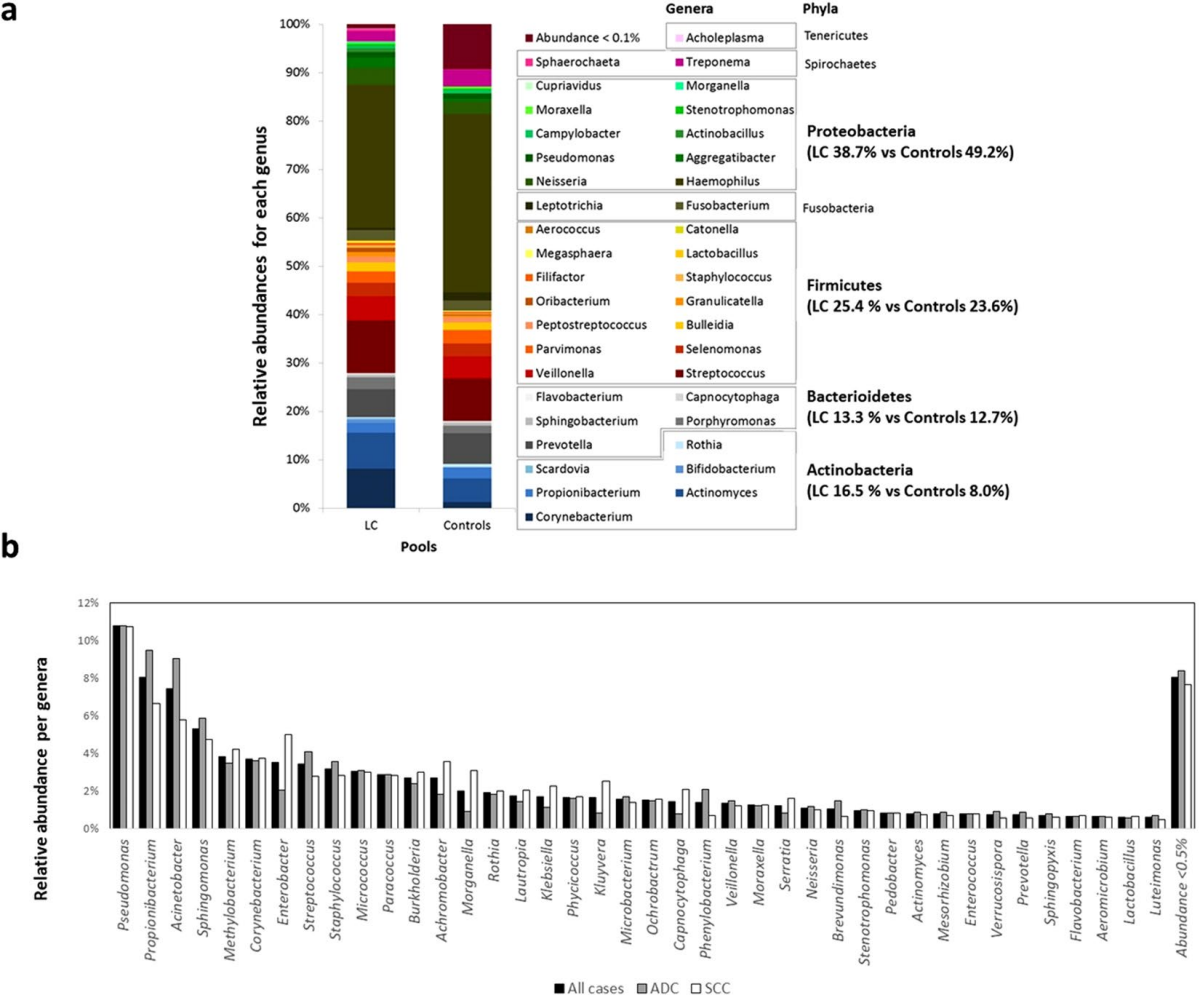
Characterization of lung cancer (LC) microbiota. (**a**) Relative abundance at the phylum and genus levels for Portuguese cases and controls. This data corresponds to the DNA pooling of 16S rRNA amplicons (V3-V6) of 49 and 54 individual samples, respectively. (**b**) Relative abundance of genera identified among tumor sections of 1009 lung cancer cases from *The Cancer Genome Atlas* (TCGA). ADC: adenocarcinoma (N = 509). SCC: Squamous cell carcinoma (N = 500).

In our pooled sequencing approach (16S rRNA V3–V6) of cases and controls using BALF samples (Supplementary Table 1), we were able to identify a total of 11 phyla and 54 genera with relative frequencies above 0.1% (Supplementary Tables 3, 4). The prevailing phyla in our dataset were Proteobacteria, Firmicutes, Actinobacteria and Bacteroidetes (Fig. 1a), as it could be expected from previous studies^8–22^. Some variation in phyla proportions were observed between pools for Proteobacteria, (38.7% in cases *vs* 49.2% in controls; Z-score P = 0.284) and Actinobacteria (16.5% in cases *vs* 8.0% in controls; Z-score P = 0.187). Among genera, *Haemophilus* (Proteobacteria); *Streptococcus* and *Veillonella*, (Firmicutes); *Corynebacterium* and *Actinomyces* (Actinobacteria) and *Prevotella* (Bacteroidetes) were the most common in the two pools. Again, our screening agreed with former reports of lung microbiota^8–22^, disclosing only non-significant changes between pools in relative abundances of *Haemophilus* (29.5% in cases *vs* 37.5% in controls; Z-score P = 0. 390) and *Corynebacterium* (8.2% in cases *vs* 1.3% in controls; Z-score P = 0.095). Overall, bacterial communities were both fairly diverse as indicated by Shannon index at genus level (2.69 in cases *vs* 2.53 in controls). However, for the inverse Simpson, LC cases were found to be considerably more diverse than controls (7.98 *vs* 5.74, respectively).

In the TCGA series (tumor RNAseq) several discrepancies to former estimated proportions were observed. With an overall frequency of 59.4%, Proteobacteria surpassed by far the remaining phyla Actinobacteria, Firmicutes, and Bacteroidetes (23.4%, 12.0% and 4.4% respectively; Supplementary Table 5). Conversely, top genera comprised *Pseudomonas*, *Acinetobacter*, *Sphingomonas*, *Methylobacterium* and *Enterobacter* (Proteobacteria); *Propionibacterium*, *Corynebacterium*, and *Micrococcus* (Actinobacteria); and *Streptococcus* and *Staphylococcus* (Firmicutes); all with average prevalence above 3%. Taxa previously identified as abundant in 16S rRNA pooled sequencing were confirmed to be present in TCGA but at variable frequencies, ranging from 3.7% for *Corynebacterium* to 0.3% for *Haemophilus* (Fig. 1b; Supplementary Table 6). Concerning microbiota diversity, TCGA cases showed similar values to our BALF samples for Shannon index (2.86 ± 0.43) and higher statistics for inverse Simpson (11.54 ± 5.06).

### Differentiation of ADC and SCC subtypes

**Figure 2:**
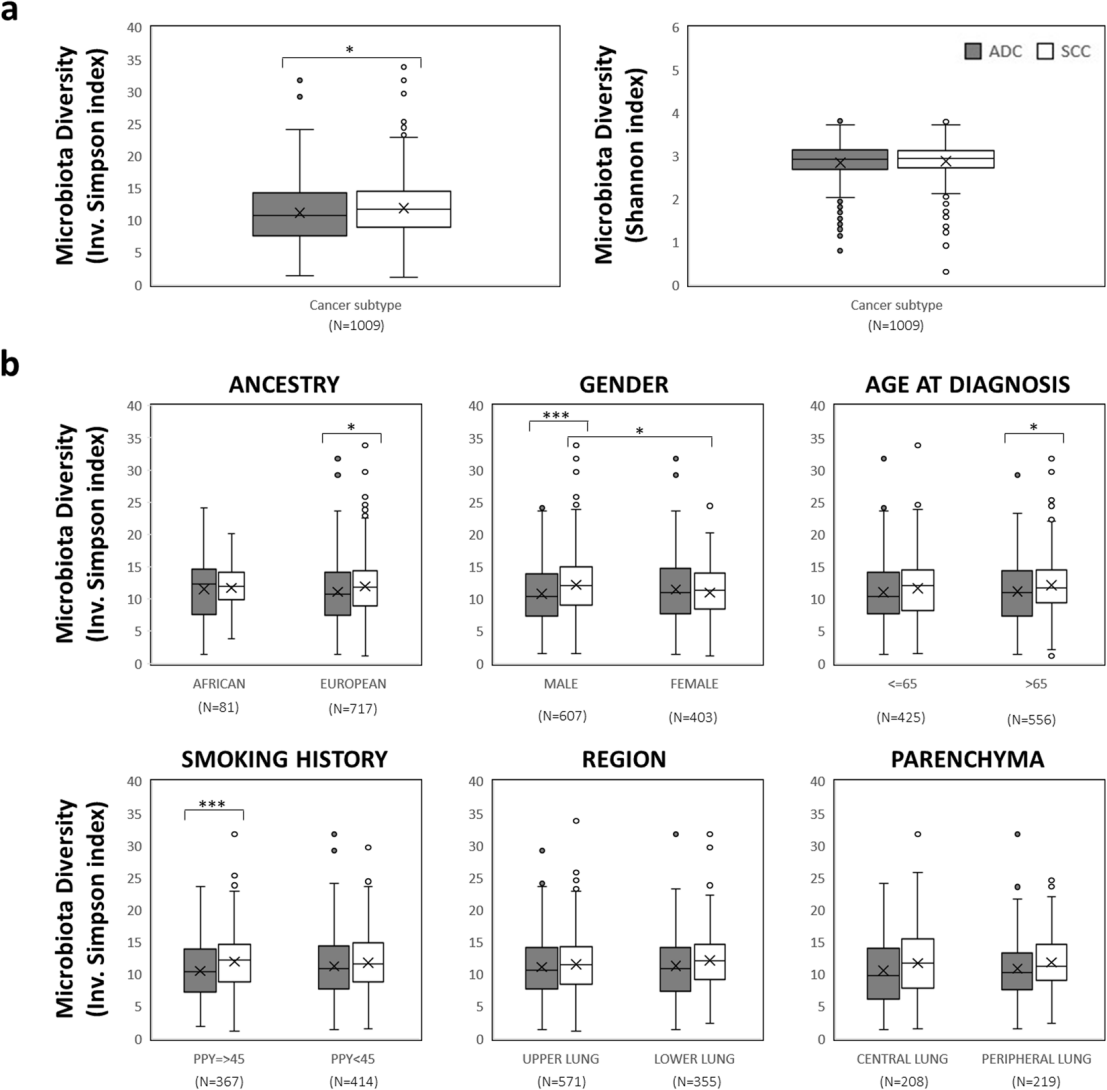
Alpha diversity of lung microbiota from lung cancer (LC) cases of *The Cancer Genome Atlas* (TCGA). (**a**) Inverse Simpson and Shannon indexes for LC cases grouped by histological subtype. (**b**) Inverse Simpson index of LC subtypes grouped according to different clinical variables available at TCGA database (ancestry, gender, age at diagnosis, smoking history, lung region and lung parenchyma). Welch’s t-test was used to access statistical significance of pairwise comparisons (*P-value < 0.05; **P-value < 0.01, ***P-value < 0.001). ADC: adenocarcinoma. SCC: Squamous cell carcinoma.

The availability of clinical parameters for TCGA cases allowed an in-depth analysis of possible factors affecting lung microbiota. Aside from some variability in genera abundance per cancer subtype (Fig. 1b), we found that SCC tends to show higher diversity than ADC as indicated by inverse Simpson (Fig. 2a). This difference seems to be correlated with European ancestry, male gender, heavy smoking (PPY >45) and older ages at the time of diagnosis (>65 years) (Fig. 2b). Yet, we could not detect any effect on microbiota diversity when considering tumor localization, upper or lower lung and central or peripheral parenchyma (Fig. 2b).

We also used TCGA dataset to test the hypothesis of a loss of microbiota diversity along with disease progression. Nevertheless, no differences were observed across pairwise comparisons of cancer stages (I, II and III + IV) or COPD presence and absence. Stratification by cancer subtype did not alter the results (Supplementary Fig. 1).

**Figure 3:**
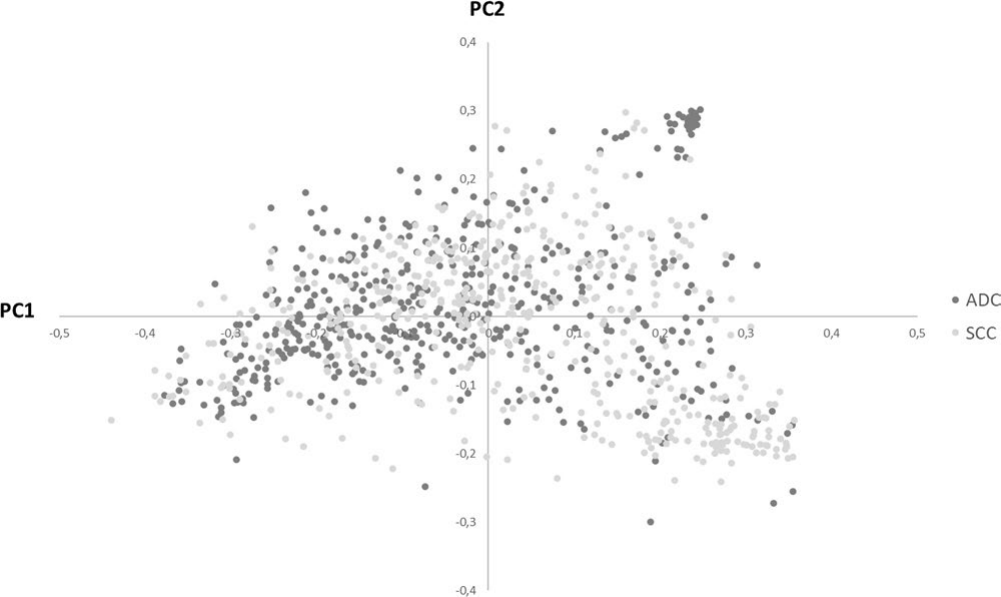
Beta diversity of lung microbiota from lung cancer (LC) cases of *The Cancer Genome Atlas* (TCGA). The Principal Coordinates Analysis (PCoA) plot was generated using weighted UniFrac distances. ADC: adenocarcinoma. SCC: Squamous cell carcinoma.

To evaluate the similarity of microbiota profiles weighted UniFrac distances were calculated. As plotted in PCoA (Fig. 3), LC communities are quite variable across samples and overlap between ADC and SCC. Nonetheless, some individual profiles appear to cluster and to be correlated with either ADC (upper right quadrant) or SCC (lower right quadrant). No other variable was found to aggregate groups of cases (results not shown).

**Figure 4:**
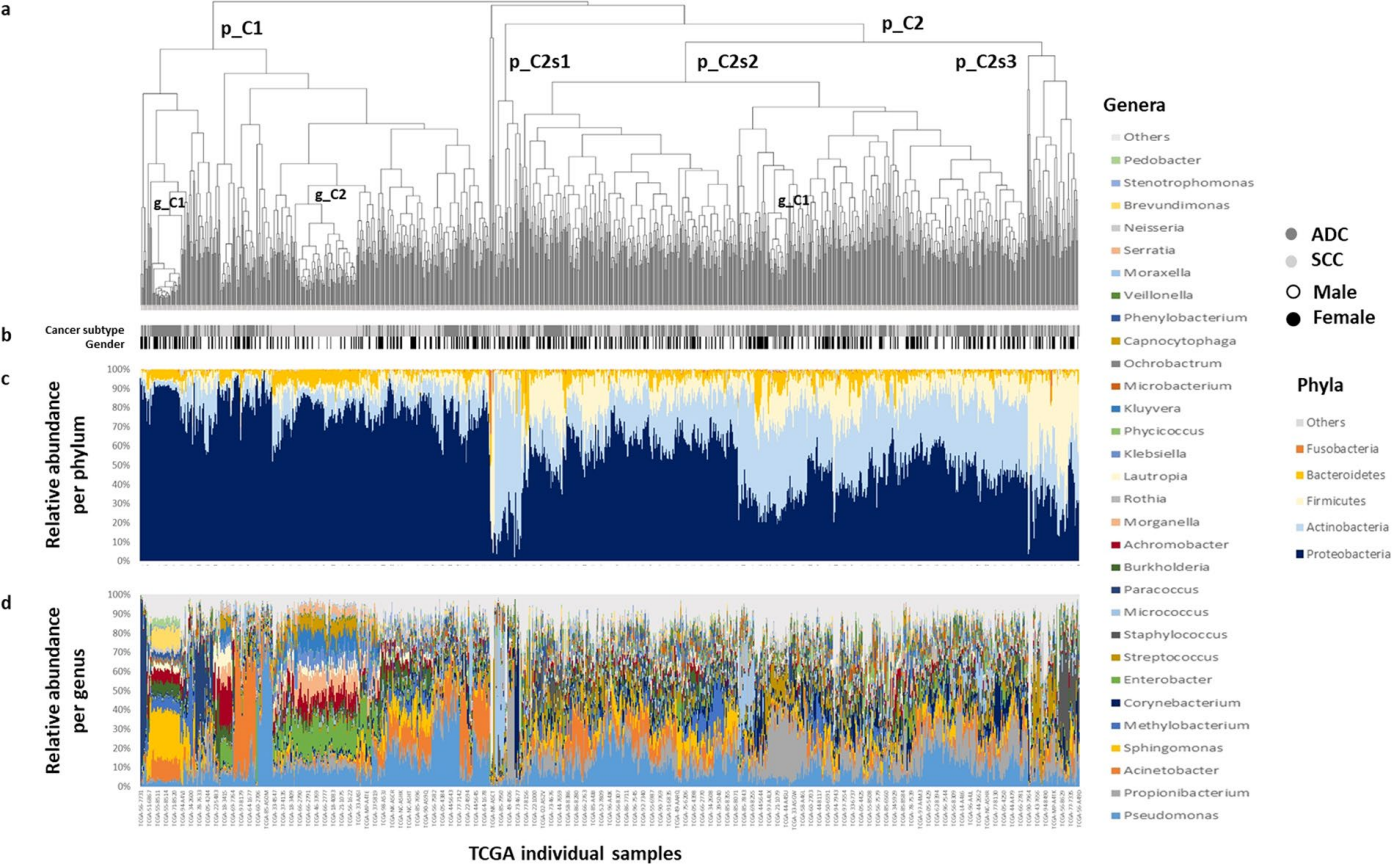
Bacterial communities of lung cancer (LC) cases from *The Cancer Genome Atlas* (TCGA). (**a**) Hierarchical clustering of LC cases built using weighted UniFrac distances and complete linkage method. Major clusters identified are indicated in the tree. (**b**) Schematic representation of LC subtype and gender variables available for all samples. (**c**) Phyla relative abundance per each sample. (**d**) Genera relative abundance per each sample. Less frequent taxa are grouped in a single category and labeled as “Others”. ADC: adenocarcinoma. SCC: Squamous cell carcinoma.

Then, to gain a better insight into LC microbiota profiles we performed a hierarchical clustering of TCGA samples, followed by a graphic display of their lung communities at two taxonomic ranks - phylum and genus (Fig. 4a–d). In the phylum analysis some heterogeneity among cases could be already witnessed, as indicated by upper tree clusters and subclusters. Higher abundances of Proteobacteria were connected with a first cluster (p_C1) and a second one (p_C2) could be divided into three major subclusters. Basically, these diverged in the relative proportions of common phyla: p_C2s1 was dominated by Actinobacteria; p_C2s2 had balanced frequencies of Proteobacteria, Actinobacteria, Firmicutes and Bacteriodetes and p_C2s3 was Firmicutes enriched.

The analysis at the genus level depicted a larger complexity of lung microbiota, where cases often clustered into small groups showing long terminal branches. Notably, inside p_C1 two clusters contrasted with the remaining tree by their shorter terminal branches (p_C1/g_C1 and p_C1/g_C2; Fig. 4a). Whereas p_C1/g_C1 could be characterized by a community composed by prevalent genera such as *Sphingomonas*, *Brevundimonas*, *Acinetobacter* and *Methylobacterium*; p_C1/g_C2 could be defined by *Enterobacter*, *Morganella*, *Kluyvera* and *Capnocytophaga*. Interestingly, p_C1/g_C1 contained only ADC cases (N = 32), all of them located in the upper right quadrant of PCoA plot (Supplementary Fig. 2). In contrast, p_C1/g_C2 included essentially SCC cases (89 in 94), this turn corresponding to the plot lower right quadrant (Supplementary Fig. 2). In the p_C2s2 a single cluster emerged as less heterogeneous (p_C2s2/g_C1), in this instance, this could be correlated with high frequencies of *Propionibacterium* and mostly linked to ADC cases (32 in 42).

**Figure 5:**
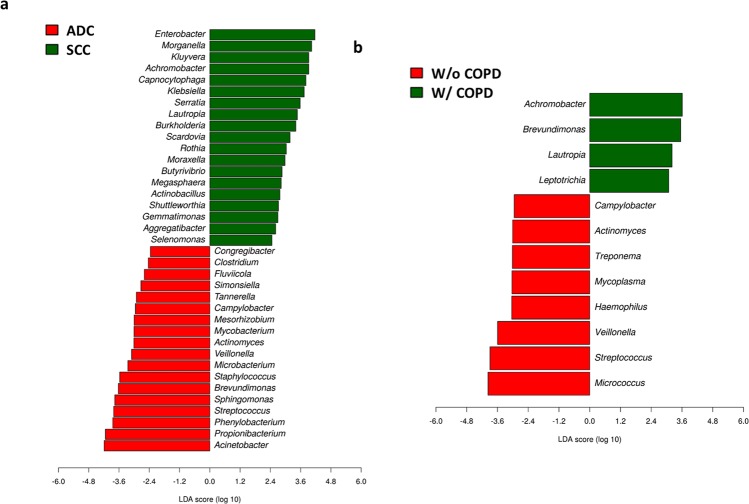
Microbial differentiation of* The Cancer Genome Atlas* (TCGA) cases according to disease status and linear discriminant analysis (LDA) effect size (LEfSe) algorithm. (**a**) lung cancer subtype. (**b**) COPD co-morbidity presence or absence. ADC: adenocarcinoma (N = 509). SCC: Squamous cell carcinoma (N = 500). W/o COPD: cases without COPD (N = 164); W/COPD: cases with COPD (N = 110).

In the LEfSE analysis of ADC and SCC cases a total of 37 genera were detected to display contrasting correlations between LC subtypes (Fig. 5a). Precisely, for ADC the genera with higher LDA scores (>3.5) and extreme P-values (P < 5 × 10^−8^) were *Acinetobacter*, *Propionibacterium*, *Phenylobacterium*, *Brevundimonas* and *Staphylococcus*. On the other hand, for SCC the genera fitting such requirements were *Enterobacter*, *Serratia*, *Kluyvera*, *Morganella*, *Achromobacter*, *Capnocytophaga* and *Klebsiella* (Supplementary Table 7). Interestingly, most of these bacteria could be correlated with previously identified clusters - p_C1/g_C1, p_C1/g_C2 and p_C2s2/g_C1. A similar approach was used to address a possible contribution of bacteria into COPD, as a common co-morbidity to both ADC and SCC (Fig. 5b). Among the 12 taxa identified *Achromobacter* was the one most strongly correlated with airflow obstruction (LDA scores >3.5 and P-values ≤0.010; Supplementary Table 7).

### Bacterial communities as prognostic biomarkers

**Figure 6:**
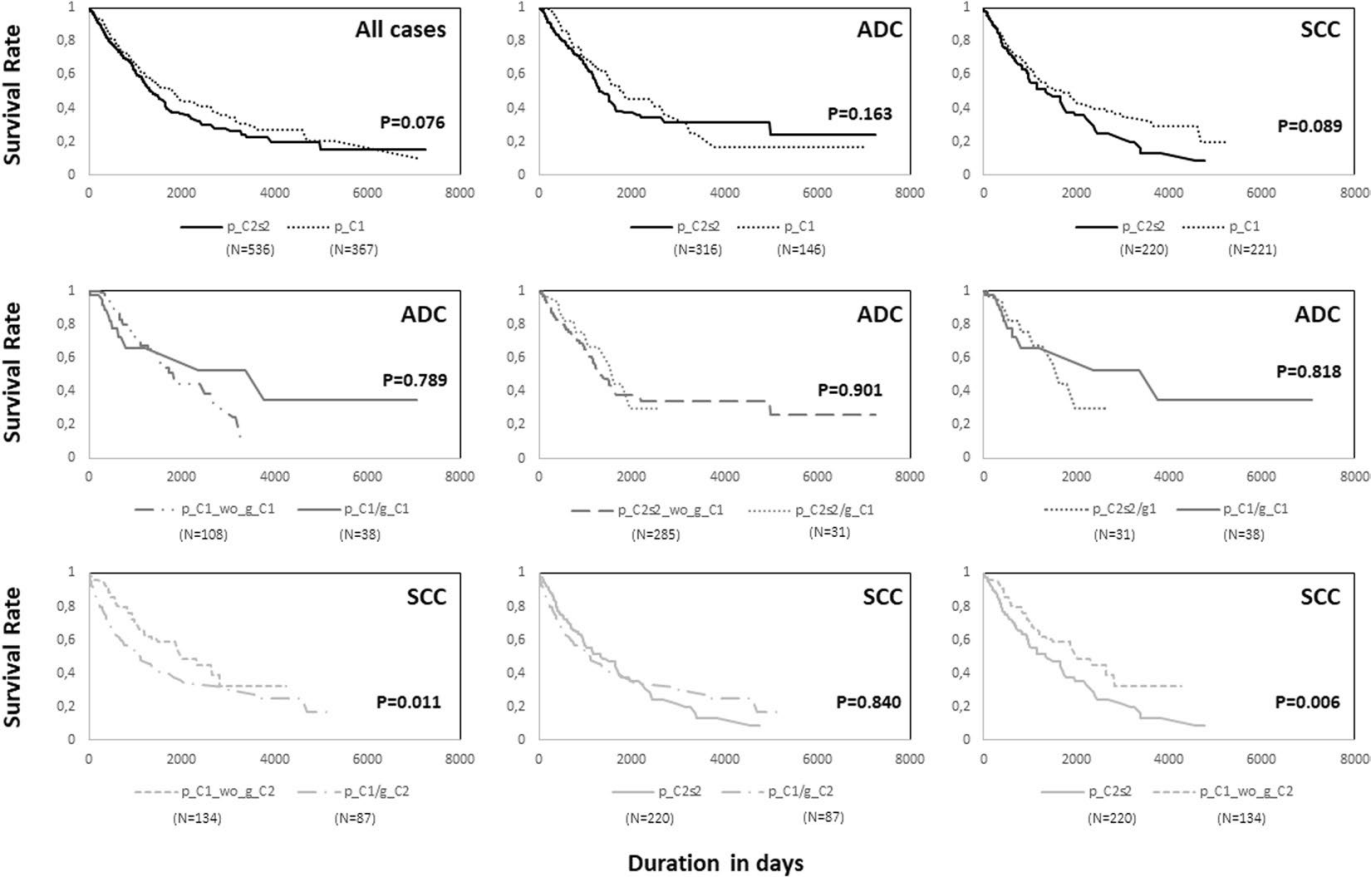
Survival plots of *The Cancer Genome Atlas* (TCGA) cases organized according to different microbial clusters identified and lung cancer subtypes. ADC: adenocarcinoma. SCC: Squamous cell carcinoma.

To investigate if our findings could have clinical potential, especially in a better stratification of LC cases, we compared the survival curves of previously identified clusters. In a first step, no significant differences were detected between p_C1 (Proteobacteria dominated) and p_C2s2 (intermediate abundances of common phyla), not even when separated by ADC and SCC. Yet, in the global comparison, and among SCC cases p_C1 cluster appears to be associated with a slower decay of survival rates (P = 0.076 and P = 0.089, respectively; Fig. 6). Several analyses were performed also in ADC, for p_C1/g_C1 (*Acinetobacter/Brevundimonas* community), p_C2s2/g_C1 (*Propionibacterium* community) and other cases, but all failed to reach compelling results possibly due to their low sample sizes. On the other hand, among SCC the p_C1/g_C2 cluster (*Enterobacter* community) was found to departure from the remaining p_C1 cases with a worse survival (P = 0.011), closer to the one observed in p_C2s2 cluster. Still, the strongest divergence in SCC survival rates was observed for non-p_C1/g_C2 (Proteobacteria dominated without *Enterobacter* community) and p_C2s2 (P = 0.006; Fig. 6). Interestingly, p_C1/g_C2 was the cluster associated with the highest mortality rate during follow-up (approximately 5000 days’ maximum for SCC and 7500 days for ADC), and the one correlated with an increased number of deaths in tumor free patients (Table 1). Conversely, non-p_C1/g_C2 was shown to display a reduced mortality even when the disease progressed negatively after primary therapy and a period of complete remission (Table 1). The small size of p_C1/g_C1 and p_C2s2/g_C1 clusters prevented in ADC an accurate evaluation of the impact of these well-defined communities in patient outcome (Table 1).

**Table 1:** Patient follow-up data across different bacterial communities.

Cancer subtype	Follow-up variables	Bacterial communities				P-values
**Adenocarcinoma** (**ADC**)		1: p_C1_g_C1	2: p_C1_wo_g_C1	3: p_C2s2/g_C1	4: p_C2s2_wo_g_C1	
	**Mortality**					
	***Total Deaths***	0.289 (11/38)	0.333 (36/108)	0.355 (11/31)	0.379 (108/285)	1 *vs* 2: P = 0.6896; 1 *vs* 3: P = 0.6106; 1 *vs* 4: P = 0.3709; 2 vs 3: P = 0.8321; 2 *vs* 4: P = 0.4147; 3 *vs* 4: P = 0.8476.
	***Days to death***					
	Mean ± SD	725.7 ± 927.0	1072.7 ± 744.8	947.5 ± 779.5	699.4 ± 652.3	
	Median	434	880	737	553.5	
	***Tumor free***					
	Number of Deaths	0.056 (1/18)	0.133 (6/45)^d^	0 (0/15)	0.095 (13/137)	1 *vs* 2: P = 0.6621; 1 *vs* 3: P = 1; 1 *vs* 4: P = 1; 2 *vs* 3: P = 0.3214; **2** ***vs*** **4**: **P** = **0**.**0338**; 3 *vs* 4: P = 0.3655.
	***With tumor***					
	Number of Deaths	0.500 (4/8)	0.563 (18/32)	0.625 (5/8)	0.674 (58/86)	1 *vs* 2: P = 1; 1 *vs* 3: P = 1; 1 *vs* 4: P = 0.4377; 2 *vs* 3: P = 1; 2 *vs* 4: P = 0.2848; 3 *vs* 4: P = 1.
	**Primary therapy outcome**					
	Complete remission	0.828 (24/29)	0.646 (51/79)	0.958 (23/24)	0.784 (171/218)	1 *vs* 2: P = 0.0983; 1 *vs* 3: P = 0.2044; 1 *vs* 4: P = 0.8086; **2** ***vs*** **3**: **P** = **0**.**0018**; **2** ***vs*** **4**: **P** = **0**.**0227**; 3 *vs* 4: P = 0.0555.
**Squamous Cell Carcinoma** (**SCC**)		**1**: **p_C1/g_C2**	**2**: **p_C1_wo_g_C2**		**3**: **p_C2s2**	
	**Mortality**					
	***Total Deaths***	0.621 (54/87)	0.336 (45/134)		0.441 (97/220)	**1** ***vs*** **2**: **P** < **0**.**0001**; **1** ***vs*** **3**: **P** = **0**.**0053**; 2 *vs* 3: P = 0.0575
	***Days to death***					
	Mean ± SD	921.4 ± 1144.5	868.2 ± 695.179		808.5 ± 823.4	
	Median	494.5	645.0		506.0	
	***Tumor free***					
	Number of Deaths	0.421 (16/38)	0.132 (9/68)		0.181 (20/110)	**1** ***vs*** **2**: **P** = **0**.**0015**; **1** ***vs*** **3**: **P** = **0**.**0045**; 2 *vs* 3: P = 0.4131
	***With tumor***					
	Number of Deaths	0.941 (16/17)	0.625 (15/24)		0.829 (34/41)	**1** ***vs*** **2**: **P** = **0**.**0281**; 1 *vs* 3: P = 0.4151; 2 *vs* 3 P = 0.0798
	**Primary therapy outcome**					
	Complete remission	0.795 (31/39)	0.913 (84/92)		0.828 (125/151)	1 *vs* 2: P = 0.0795; 1 *vs* 3: P = 0.6425; 2 *vs* 3: P = 0.0853

Significant p-values for Fisher’s exact test (p < 0.05) are shown in bold.

## Discussion

In this study, we performed a characterization of LC microbiota using two distinct datasets and methodological approaches: a pooled sequencing of 16S rRNA in BALF samples from Portuguese cases and controls; and a surveying of bacterial RNAseq reads made available through TCGA, for which tumor sections of hundreds of patients were collected. The main advantage of the first approach was to provide a preliminary and raw overview of lung microbiota at very low cost. However, this pooled approach has several limitations starting by its inability to address inter-individual variability and to accurately pinpoint bacterial communities to each individual. Another weakness is related to sample heterogeneity, which contains several cases lacking a complete histological classification and controls that include manly subjects with other pathologies. At last, surveying 16S rRNA can be considered also a shortcoming, once it is expected to introduce some ascertainment bias in taxa identification. This caveat is attributed, on one hand, to the differential annealing affinities of universal primers used in 16S rRNA amplification, and on the other, to the distinct resolving power of covered hypervariable regions^27,31^. Conversely, in the second approach, we could benefit from a larger cohort of ADC and SCC cases, for which detected RNAseq reads are more likely to represent an accurate composition of lung microbiota. The unique disadvantage of this strategy is that in order to maximize efficiency in mapping bacterial reads, we provided a database of reference genomes^28^. This was built using taxa identified in our 16S rRNA survey, combined with HMP data and published elsewhere^8–22^.

Overall, Proteobacteria emerged as the predominant LC phylum, a trend captured also in a large sample of cancer patients for which non-malignant tissue sections were collected^17^. Until now, increased frequencies of Proteobacteria were mostly correlated with asthma, COPD exacerbations and advanced COPD stages^8,9,32,33^. However, given current findings a Proteobacteria enrichment could also be a feature of cancerous lungs. In-depth surveys uncovered distinctive scores of Proteobacteria defining two major clusters: a first one truly dominated by Proteobacteria (p_C1); and another one displaying intermediate frequencies of Proteobacteria, Actinobacteria, Firmicutes and Bacteroidetes (p_C2s2). Altogether, these results are suggestive of substructure in lung microbiota that as far as we could investigate is not correlated with cancer subtype, or any other evaluated clinical variable.

Also, in a global perspective, *Pseudomonas*, *Streptococcus*, *Staphylococus*, *Veillonella* and *Moraxella* were identified among the top rank bacteria of cancer cases fitting the so-called lung core microbiome^8–22^. Nevertheless, individual distributions showed a different scenario, in which cases are generally quite divergent in their microbial composition. The exceptions to this rule are three specific communities displaying remarkable links to LC subtypes: *Brevundimonas/Acinetobacter* (p_C1/g_C1) for ADC; *Enterobacter* (p_C1/g_C2) for SCC, and *Propionibacterium* (p_C2/g_C1) for ADC.

Most impressively, p_C1/g_C2 cluster could be related with an overall poor survival if comparing SCC cases within p_C1 group. Furthermore, p_C1/g_C2 was characterized by several Enterobacteriaceae (*Enterobacter*, *Morganella*, *Serratia*, *Klebesiela and Kluyvera*), a taxon with recognized pathogenic potential causing airway infections in COPD^34^, colonizing bronchi of LC patients^35^ and underlying nosocomial infections with resistance to antimicrobial molecules^36^. This cluster comprised also *Achromobacter*, another multidrug resistant microbe previously found in airway infections of cystic fibrosis patients and among subjects with solid malignancies^37^.

Moreover, as gram-negative bacteria, Enterobacteriaceae and *Achromobacter* synthetize lipopolysaccharides capable of stimulating host inflammatory responses. In this respect, Enterobacteriaceae overgrowth has been described as a key event of gut dysbiosis in obesity, Crohn’s disease and colorectal cancer^37,38^. In asthma and cystic fibrosis there is also a growing body of evidence for a negative effect of Enterobacteriaceae^39,40^. Therefore, a contribution of p_C1/g_C2 community to an enhanced pro-inflammatory cancer microenvironment seems like a plausible hypothesis, once it is reported to foster tumorigenesis and promote lung cells malignant transformation^4,41^. This rational is supported by reports for a worse LC prognosis when patient bronchi are colonized by Enterobacteriaceae^35^.

Nonetheless, p_C1/g_C2 cluster was also linked with an increased mortality in absence of any tumor, which suggests an increased risk of this group to other non-cancer complications. Indeed, several studies already reported diverse pulmonary infections, septicemia and enhanced death rates after cancer resection and chemotherapy in LC subjects carrying potential pathogenic microorganisms in their airways^35,42,43^. Although we were unable to rigorously address the impact of p_C1/g_C2 community in the health status of a group of individuals probably debilitated by advanced age, co-morbidities (e.g. COPD, cardiovascular disease, diabetes, *etc*.) and inclusively cancer treatment, our findings advocate for a differentiated medical intervention in these patients, namely in the selection of antimicrobial therapies.

Still, the overall survival of p_C1/g_C2 does not differ from p_C2s2 cluster, which advances Actinobacteria, Firmicutes and/or Bacteriodetes as additional risk factors in SCC possibly through similar mechanisms of cancer progression. On the contrary, non-p_C1/g_C2 group appears to somehow tolerate new tumor events, which leads to a decoupling of its survival curve from the rapid decline of p_C1/g_C2, and from the continuous decrease of p_C2s2.

Less remarkable findings were obtained for *Brevundimonas/Acinetobacter* (p_C1/g_C1) community, which did not diverge from other cases concerning ADC outcomes. However, if compared with SCC p_C1/g_C2 and p_C2s2 groups those disclosed a trend for extended survival or lifetime prognosis.

We also provide additional pieces of information for a burden of microbiota in pulmonary disease. In the TCGA cohort, cases showing COPD co-morbidity were linked to *Achromobacter*, thus highlighting a negative effect of this taxon in SCC and in airflow obstruction. However, COPD was not associated to any identified bacterial community in particular, nor to augmented prevalence of *Moraxella* and *Haemophilus* genera as frequently observed among these patients^8,12,22^. Noticeably, only 27% of cases were presented with lung function tests, and for those with COPD (11%) their majority was classified as mild or moderate cases (FEV > 50%; GOLD 1-2 stages). These features could explain the similar alpha diversity scores obtained for patients with or without COPD, once previous reports for a microbiota loss were centered in advanced cases^22^ (for opposite results^19,40^). More striking is the lack of differentiation across LC stages if taking into account former reports for a loss of diversity between tumor and non-tumor samples^17,18^. Nonetheless, there is no proof so far for a gradual decline of microbiota with cancer progression. In contrary, previous works uncovered higher alpha diversity values in advanced cases (IIIB and IV) than in earlier disease stages^18^. In our study, SCC cases were in average more diverse than ADC, a result that can be related to a heavier smoking load of these patients since cigarette and air pollutants were found to positively affect airway microbiota richness^17,23^. However, other unknown factors must play a role in LC bacterial colonization to explain the significant differences observed between SCC and ADC in heavy smokers. Moreover and consistently with a recent study carried out in mild to moderate COPD^12^, we also reported similar diversity levels across distinct lung regions (bronchial and peripheral lung), contradicting earlier findings for a microbiota differentiation in disparate lung anatomical regions^10^.

To our knowledge this work represents the largest scrutiny of LC microbiota. Shortly, we uncovered a predominance of Proteobacteria among cancerous lungs a feature shared with other airway disorders. However, Proteobacteria abundance is not universal and rather dictates a microbiota substructure independently of LC subtype, COPD co-morbidity, smoking history, age or lung region. In SCC, we found evidence for a differential effect of bacterial communities in patient survival, particularly when stratified into an Enterobacteriaceae cluster. Given that this taxon has documented complications in pulmonary illnesses, we proposed a contribution of this cluster to an inflammatory cancer microenvironment, as well as, for other post-operative and/or therapeutic non-cancer complications. Finally, we believe that the discovery of such well-defined communities may shed light into bacteria as promising LC biomarkers for patient stratification and as future prognostic tools or even as therapeutic targets.

## Supplementary information

Supplementary Dataset 1

Supplementary Dataset 2

## Data Availability

The data used in this study is included in this manuscript and in supplementary material files. Additional files used to generate data analysis are available from the corresponding author on reasonable request.
