# Supplementary material for: Profiling of lung microbiota discloses differences in adenocarcinoma and squamous cell carcinoma
Source: Sci Rep. 2019 Sep 6;9:12838. doi: 10.1038/s41598-019-49195-w (PMC6731246; doi:10.1038/s41598-019-49195-w)
Supplement: Supplementary file 1 — Supplementary Dataset 1 [file 41598_2019_49195_MOESM1_ESM.pdf]

Profiling of lung cancer microbiota discloses differences in adenocarcinoma and squamous cell carcinoma

Sílvia Gomes, Bruno Cavadas, Joana Catarina Ferreira, Patrícia Isabel Marques, Catarina Monteiro, Maria Sucena, Catarina Sousa, Luís Vaz Rodrigues, Gilberto Teixeira, Paula Pinto, Tiago Tavares de Abreu, Cristina Bárbara, Júlio Semedo, Leonor Mota, Ana Sofia Carvalho, Rune Matthiesen, Luísa Pereira, Susana Seixas*

*Corresponding author: sseixas@ipatimup.pt

SUPPLEMENTARY FIGURES

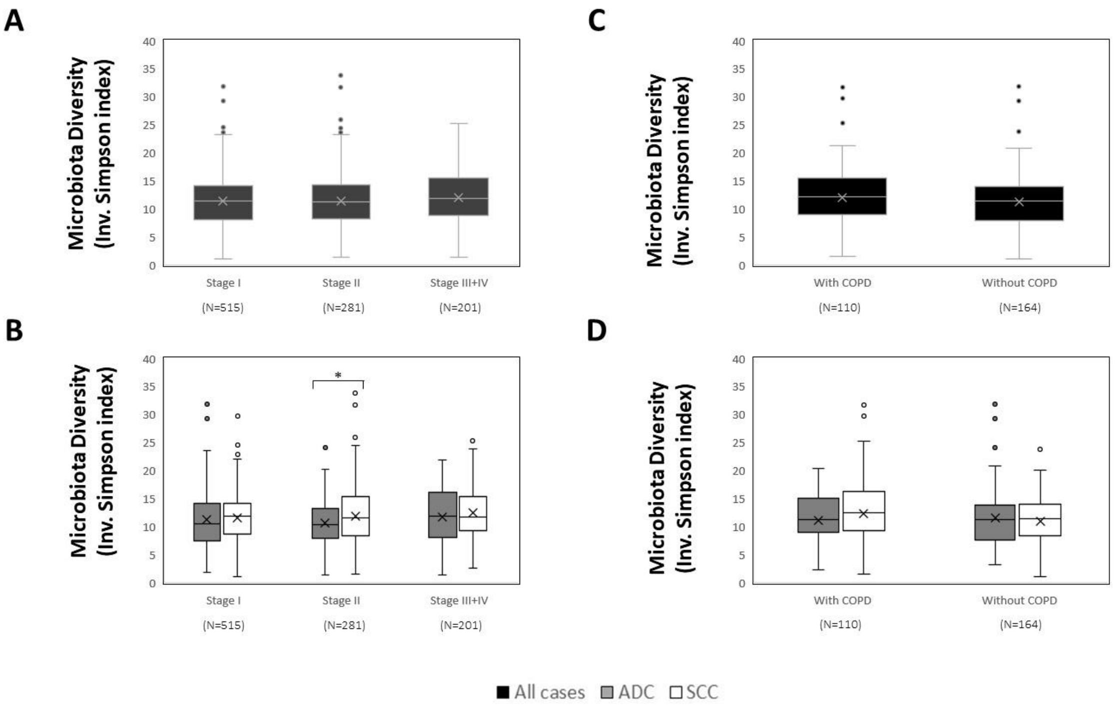

Supp. Fig. 1: Lung Microbiota diversity (inverse Simpson index) from The Cancer Genome Atlas (TCGA) cases according to disease status. A) Lung cancer (LC) stage. B) LC stage within histological subtype. C) COPD co-morbidity among LC cases. D) COPD co-morbidity within LC subtype. Welch's t-test was used to access statistical significance of pairwise comparisons (*P-value <0.05). ADC: adenocarcinoma. SCC: Squamous cell carcinoma.

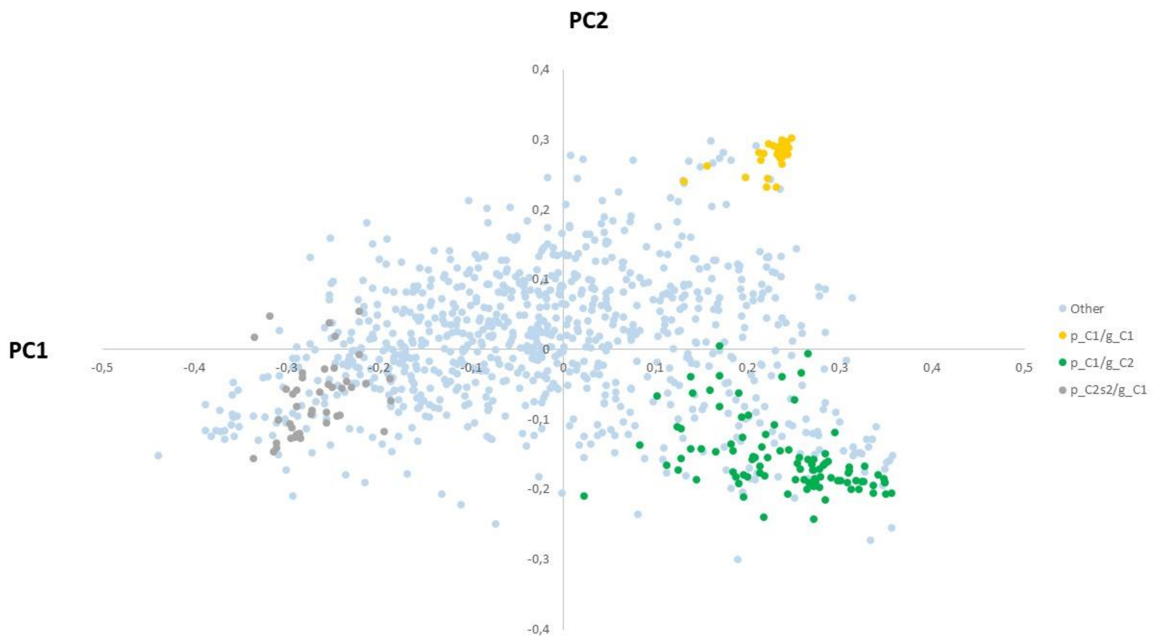

Supp. Fig. 2: Coordinates Analysis (PCoA) plot highlighting major clusters identified among The Cancer Genome Atlas (TCGA) cases.
